# Dual *vs*. isolated anti-Ro antibody positivity in rheumatoid arthritis

**DOI:** 10.3389/fimmu.2025.1661334

**Published:** 2025-08-18

**Authors:** Yan Ma, Chaoyu Gu, Qianqian Li, Liangjing Lu

**Affiliations:** Department of Rheumatology, Renji Hospital, Shanghai Jiao Tong University School of Medicine, Shanghai, China

**Keywords:** rheumatoid arthritis (RA), anti-Ro52 antibody, anti-Ro60 antibody, difficult-to-treat rheumatoid arthritis (D2T-RA), anti-Ro antibody subtyping

## Abstract

**Objective:**

This study aimed to evaluate the clinical and immunological significance of anti-Ro52/TRIM21 and anti-Ro60/SSA antibodies in rheumatoid arthritis (RA), particularly the association of dual antibody positivity with disease severity, systemic manifestations, and therapeutic resistance.

**Methods:**

We conducted a cohort study involving 670 RA patients, stratified into four groups according to anti-Ro52 and anti-Ro60 antibody status: Ro52+/Ro60+, Ro52+/Ro60−, Ro52−/Ro60+, and Ro52−/Ro60−. Clinical characteristics, disease activity scores (DAS28-ESR, DAS28-CRP), systemic complications, and treatment responses were compared among groups. Multivariate logistic regression models identified independent predictors of difficult-to-treat RA (D2T-RA).

**Results:**

Patients with dual Ro52+/Ro60+ positivity exhibited significantly higher disease activity (median DAS28-ESR: 4.97 *vs*. 4.39, p = 0.002), worse functional status (median HAQ-DI: 0.88 *vs*. 0.63, p = 0.001), and increased systemic complications, notably interstitial lung disease (OR = 4.14, 95% CI: 1.71–10.68, p = 0.002) and hematologic involvement (OR = 2.50, 95% CI: 1.02–6.19, p = 0.044), compared to antibody-negative patients. Dual antibody positivity independently predicted an increased risk of developing D2T-RA (OR = 4.05, 95% CI: 1.58–11.09, p = 0.004). Conversely, patients with isolated Ro60 positivity exhibited lower IgG levels, fewer systemic complications, and reduced reliance on biological therapies, indicating a less severe disease phenotype.

**Conclusion:**

Anti-Ro antibody subtyping effectively identifies distinct clinical and immunological RA subgroups. Patients with isolated Ro60 antibody positivity display a relatively less severe clinical profile compared to those with dual antibody positivity, highlighting the importance of specific antibody profiles in guiding personalized clinical management and therapeutic decision-making.

## Introduction

Anti-Ro60 (SSA) and anti-Ro52 (TRIM21) antibodies target distinct ribonucleoprotein (RNP) complexes and are established biomarkers in Sjögren’s disease (SjD) and systemic lupus erythematosus (SLE) (1–3). These autoantibodies recognize antigens with divergent cellular roles: Ro60/SSA binds to noncoding RNAs called hY-RNA and allows the degradation of misfolded

RNA in addition to molecular chaperones (4), while Ro52/TRIM21 is a protein with E3 ligase activity that modulating interferon (IFN) signaling and apoptosis (5, 6). Although their coexistence or isolated positivity may reflect distinct immunopathological mechanisms, research has predominantly focused on anti-Ro52 due to its association with interstitial lung disease (ILD) in connective tissue diseases (CTDs), particularly in antisynthetase syndrome (ASS) (7), systemic sclerosis (SSc) (8, 9), Sjögren’s disease (SjD) (10, 11), and mixed connective tissue disease (MCTD) (12). Emerging evidence suggests that dual positivity for anti-Ro52/TRIM21 and anti-Ro60/SSA antibodies correlates with severe phenotypes in SjD (13). Similar findings have also been observed in patients with SLE (14, 15), yet the clinical implications of these antibody profiles in rheumatoid arthritis (RA) remain under explored.

Anti-Ro/SS-A antibodies, though diagnostic for SjD, are detected in 3–15% of RA patients (16). Notably, while the prevalence of anti-Ro52 in RA is lower compared to SjD and SLE, studies report that anti-TRIM21 positivity in pre-RA females frequently coexists with anti-Ro60 positivity (17). Difficult-to-treat rheumatoid arthritis (D2T RA) is a recently recognized phenotype of RA characterized by persistent disease activity despite the use of multiple advanced therapies (18). However, to date no reliable biomarkers have been established to predict the development of D2T RA, reflecting the limited understanding of factors driving refractoriness. Autoantibody profiles are central to RA classification and prognosis. Rheumatoid factor (RF) (19) and anti-citrullinated protein antibodies (ACPA) (20) are well-known predictors of severe disease, yet they are not specifically linked to treatment outcomes. Attention has therefore turned to other autoantibodies that might delineate high-risk subsets. Recent studies indicate that RA patients positive for anti-Ro/SSA antibodies represent a distinct clinical subset with higher disease activity, more frequent extra-articular features (such as sicca symptoms), and reduced responsiveness to certain DMARDs (21). In particular, the presence of anti-Ro antibodies has been linked to inadequate responses to TNF inhibitors and methotrexate, suggesting that antibody subtypes could play a role in risk stratification and guiding therapy selection (16). Given this background, we hypothesized that anti-Ro antibody status (in particular, dual positivity for both Ro52 and Ro60 *vs*. isolated single positivity) could serve as a predictive marker for D2T RA. In this study, we investigated the clinical significance of dual *vs*. isolated anti-Ro antibody positivity in RA, focusing on associations with the development of D2T RA and implications for patient risk stratification.

## Methods

### Study population

**Figure 1:**
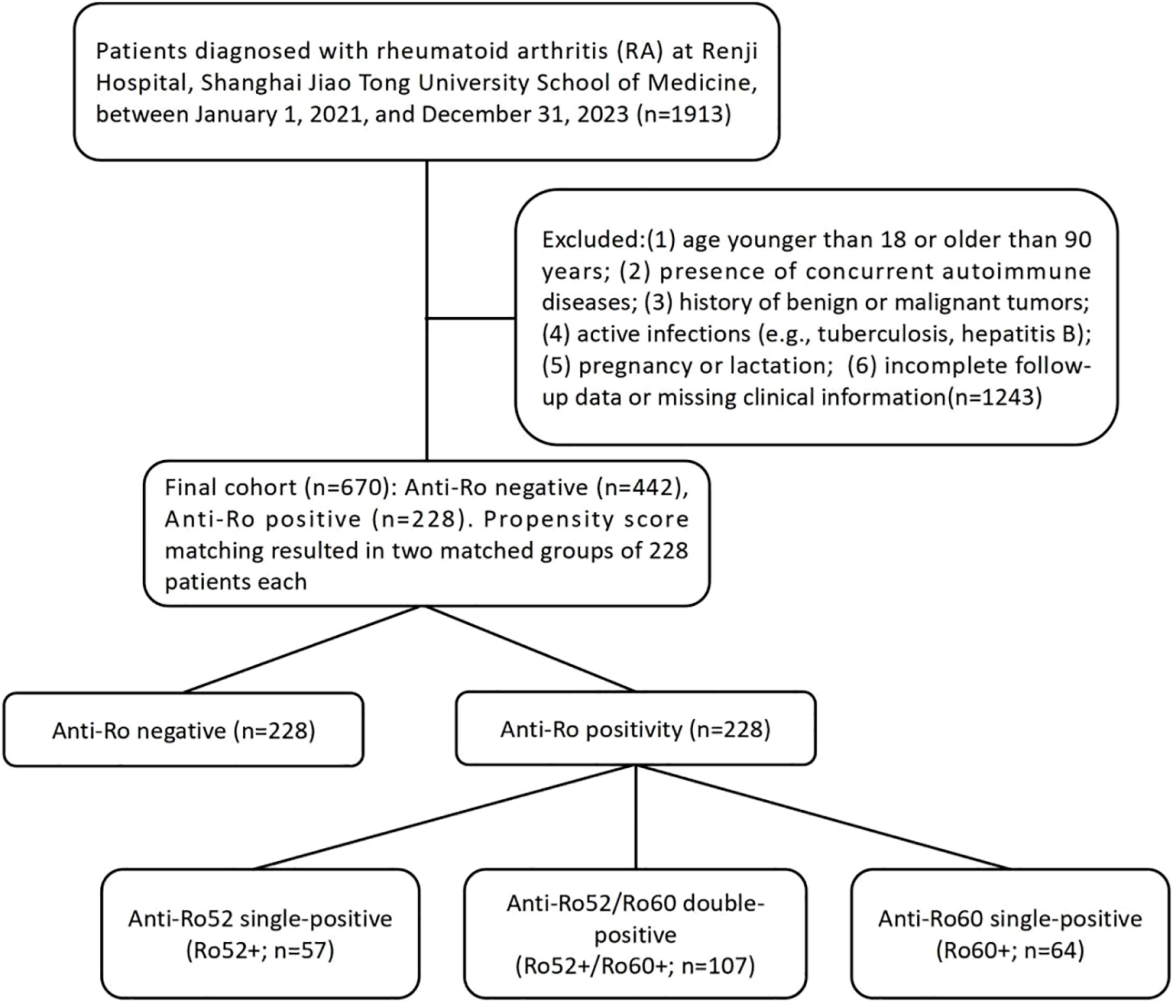
Study flowchart: patient enrollment, exclusion criteria, and stratification by Anti-Ro52/Ro60 antibody profiles in rheumatoid arthritis cohort.

This cohort study enrolled patients diagnosed with rheumatoid arthritis (RA) at Renji Hospital, Shanghai Jiao Tong University School of Medicine, between January 1, 2021, and December 31, 2023. All patients fulfilled the 2010 American College of Rheumatology/European Alliance of Associations for Rheumatology (ACR/EULAR) RA classification criteria. Exclusion criteria included the following:(1) age <18 or >90 years; (2) presence of concurrent autoimmune diseases, including rheumatoid arthritis (RA) complicated by Sjögren’s syndrome (SjD) and SjD with joint involvement, based on the 2016 ACR/EULAR classification criteria for primary Sjögren’s syndrome (total score ≥4); (3) history of benign or malignant tumors; (4) active infections, such as tuberculosis and hepatitis B; (5) pregnancy or lactation; and (6) incomplete follow-up data or missing clinical information (**Figure 1**). All enrolled participants were followed for at least one year. This study was reviewed and approved by the Ethics Committee of Renji Hospital, Shanghai Jiao Tong University School of Medicine (approval No. RA-2022-538). All participants gave written informed consent.

### Data collection

Baseline sociodemographic variables, prior medication use, autoantibody profiles (including rheumatoid factor), and disease activity were systematically recorded during the initial rheumatology consultation by senior rheumatologists. Joint involvement was assessed using the 28-tender joint count (TJC28) and 28-swollen joint count (SJC28), while extra-articular manifestations were comprehensively evaluated. Patients were guided to complete the Health Assessment Questionnaire Disability Index (HAQ-DI), and disease activity was quantified using the 28-joint Disease Activity Score based on erythrocyte sedimentation rate (DAS28-ESR), C-reactive protein (DAS28-CRP), patient global assessment (PtGA), and physician global assessment (PhGA). At each subsequent visit, data regarding medication adjustments, joint and extra-articular manifestations, and comorbidities were systematically updated. Treatment response was assessed at the 12-month follow-up.

We assessed the incidence of difficult-to-treat rheumatoid arthritis (D2T-RA) across the different antibody-defined groups. D2T-RA was defined as persistent disease activity (DAS28-ESR ≥2.6) despite adequate trials of conventional synthetic disease-modifying antirheumatic drugs (csDMARDs), as well as treatment failure with at least two biologic or targeted synthetic DMARDs (b/tsDMARDs) with distinct mechanisms of action, unless contraindications to these therapies existed.

Systemic complications were identified through patient self-reports and medical record reviews. Specific definitions included: 1) sicca symptoms: dryness of eyes or mouth; 2) Interstitial lung disease (ILD): Radiologically confirmed interstitial abnormalities on high-resolution chest computed tomography (CT), excluding alternative etiologies; 3) Hematologic involvement: Persistent leukopenia (<4.0×10^9^/L), anemia (hemoglobin <120 g/L in women, <130 g/L in men), or thrombocytopenia (<150×10^9^/L) during follow-up; 4) Renal involvement: estimated glomerular filtration rate (eGFR) <60 mL/min/1.73 m^2^ or serum creatinine >1.2 mg/dL); 5) Liver involvement: Elevated liver enzymes (ALT >1.5× upper limit of normal).

### Autoantibody measurement

All autoantibody measurements—including antinuclear antibody (ANA), rheumatoid factor (RF), anti-cyclic citrullinated peptide antibodies (ACPA), anti-Ro52, and anti-Ro60—were performed on baseline serum samples collected at patient enrollment. ANA detection was performed using indirect immunofluorescence assay (IIFA). RF and ACPA were measured using enzyme-linked immunosorbent assay (ELISA). Anti-Ro52 and anti-Ro60 antibodies were identified by immunoblotting as part of an extensive antinuclear antibody profile analysis. All assays were performed in the clinical immunology laboratory of Renji Hospital according to standardized protocols, with internal quality control to ensure reliability and consistency.

### Statistics

To minimize baseline confounding, propensity score matching (PSM) was conducted based on age, sex, age at disease onset, and body mass index (BMI), using logistic regression followed by 1:1 nearest-neighbor matching (caliper = 0.02). Continuous data are expressed as mean ± standard deviation (SD) or median (interquartile range, IQR) according to normality distribution. Group comparisons utilized independent t-tests or Mann-Whitney U tests for continuous variables, and one-way ANOVA, Welch’s, or Kruskal-Wallis tests for multiple groups as appropriate. Categorical variables were compared using chi-square, Yates’ correction, or Fisher’s exact tests, depending on cell frequencies. Ordered categorical data were analyzed via Mann-Whitney U tests, and correlations assessed using Spearman’s rank test. Multivariate logistic regression with backward stepwise elimination identified independent predictors of difficult-to-treat rheumatoid arthritis (D2T-RA), with a retention criterion of p < 0.05. Covariates were selected based on baseline group comparisons, with variables showing significant differences between the D2T-RA and non-D2T-RA groups considered for inclusion. In addition, RF and anti-CCP positivity, as well as demographic variables (such as sex and BMI), were included based on prior literature demonstrating their association with disease severity and outcomes, and to adjust for potential confounding. Model performance was assessed using receiver operating characteristic (ROC) curve analysis, and the area under the curve (AUC) was calculated to evaluate the discriminative ability of the multivariate logistic regression model. Statistical analyses were performed using SPSS v26, with statistical significance set at a two-tailed α-level of 0.05.

## Results

The study cohort consisted of 670 rheumatoid arthritis (RA) patients divided into two distinct groups based on anti-Ro antibody status: anti-Ro positive (n = 228, 34.03%) and anti-Ro negative (n = 442, 65.97%). Baseline demographic and clinical characteristics differed significantly between these groups (**Table 1**). Compared to anti-Ro negative patients, anti-Ro positive patients included fewer males (7.5% *vs*. 16%, p = 0.002), were younger (median age 45 *vs*. 53 years, p = 0.014), and had lower rates of alcohol consumption(5.7% *vs*. 12%, p = 0.008). After propensity score matching based on predefined baseline characteristics (sex, age, BMI, smoking history, and alcohol consumption), both groups were balanced at 228 patients each. Patients were further stratified into four subgroups according to their anti-Ro52 and anti-Ro60 antibody status: Ro52+/Ro60+ (n = 107, 23.46%), Ro52+/Ro60- (n = 57, 12.50%),Ro52-/Ro60+ (n = 64, 14.04%), and Ro52−/Ro60− (n = 228, 50.00%) groups.

**Table 1:** Baseline characteristics of anti-Ro positive and negative patients.

Variable	Anti-Ro negative N = 442	Anti-Ro positive N = 228	P-value
Baseline demographic			
Male	70 (16%)	17 (7.5%)	0.002
Age	53 (37-61)	45 (36-58)	0.014
BMI	22.0 (19.9-24.4)	22.5 (20.2-24.5)	0.213
Alcohol consumption	54 (12%)	13 (5.7%)	0.008
Ever Smoke	35 (7.9%)	12 (5.3%)	0.202
Course,yrs	2.09 (1.00-3.00)	2.51 (1.00-4.00)	0.003
Clinical characteristic			
WBC (×10^9/L)	6.63 (5.24-8.65)	5.91 (4.80-7.48)	<0.001
Hb (g/L)	125 (114-135)	126 (114-133)	0.894
PLT (×10^9/L)	254 (211-311)	251 (212-299)	0.464
ALT (U/L)	18 (14-27)	21 (12-31)	0.306
AST (U/L)	22 (14-38)	21 (12-43)	0.703
RF positive	319 (72%)	174 (76%)	0.249
CCP positive	368 (83%)	206 (90%)	0.013
GPI positive	67 (15%)	35 (15%)	0.948
ANA			<0.001
ANA titre=0	121 (53%)	21 (9.2%)	
0<ANA titre<=320	99 (43%)	157 (69%)	
ANA titre>320	8 (3.5%)	50 (22%)	
ESR (mm/h)	24 (13-49)	31 (18-55)	<0.001
CRP (mg/h)	6 (2-18)	4 (1-13)	0.070
IgA (g/L)	2.83 (2.17-3.59)	2.60 (1.98-3.52)	0.115
IgM (g/L)	1.37 (1.01-1.96)	1.42 (0.95-1.93)	0.770
IgG (g/L)	14.8 (12.6-17.1)	16.5 (14.2-19.5)	<0.001
C3 (g/L)	1.14 (0.99-1.30)	1.12 (0.96-1.30)	0.427
C4 (g/L)	0.26 (0.20-0.31)	0.24 (0.20-0.29)	0.117

Values are presented as median (interquartile range) for continuous variables and number (percentage) for categorical variables. Comparisons between anti-Ro negative and anti-Ro positive groups were performed using the Mann–Whitney U test for continuous variables and the Chi-square or Fisher’s exact test for categorical variables, as appropriate.Statistical significance was defined as p < 0.05.

BMI, body mass index; WBC, white blood cell count; Hb, hemoglobin; PLT, platelet count; ALT, alanine aminotransferase; AST, aspartate aminotransferase; RF, rheumatoid factor; CCP, cyclic citrullinated peptide; GPI, glucose-6-phosphate isomerase; ESR, erythrocyte sedimentation rate; CRP, C-reactive protein; IgA, immunoglobulin A; IgM, immunoglobulin M; IgG, immunoglobulin G; C3, complement component 3; C4, complement component 4.

Clinical characteristics differed notably among these antibody subgroups, particularly in inflammatory and immunological markers (**Table 2**). ESR levels were significantly higher in Ro52+/Ro60+ (median 34 mm/h), Ro52+/Ro60- (median 29 mm/h), and Ro52-/Ro60+ patients (median 27 mm/h) compared to the Ro52-/Ro60- group (median 23 mm/h; p < 0.001). IgG levels differed significantly among the subgroups, with Ro52+/Ro60− (median 17.4 g/L) and Ro52+/Ro60+ (median 17.2 g/L) patients exhibiting higher levels compared to Ro52−/Ro60+ (median 14.9 g/L) and Ro52−/Ro60− (median 14.4 g/L) patients (p < 0.001). Significant differences were observed in ANA titers (p < 0.001), with the highest prevalence of high ANA titers (>320) in the Ro52+/Ro60+ group (26%) compared to Ro52-/Ro60- group (3.5%).

**Table 2:** Baseline demographic and clinical characteristics stratified by anti-Ro52/Ro60 antibody profiles.

Variable	Ro52-/Ro60- N = 228	Ro52+/Ro60- N = 57	Ro52+/Ro60+ N = 107	Ro52-/Ro60+ N = 64	P-value
Baseline demographic					
Male, n (%)	19 (8.3%)	6 (11%)	7 (6.5%)	4 (6.3%)	0.785
Age, yrs	46 (36-59)	50 (38-58)	42 (36-56)	49 (35-62)	0.431
BMI (m/kg²)	22.5 (20.2-24.7)	23.1 (20.2-25.4)	22.4 (20.2-24.1)	22.5 (20.2-24.5)	0.680
Ever Drink, n (%)	14 (6.1%)	4 (7.0%)	6 (5.6%)	3 (4.7%)	0.961
Ever Smoke, n (%)	12 (5.3%)	5 (8.8%)	4 (3.7%)	3 (4.7%)	0.604
Onset age ≤50 yrs	157 (69%)	30 (53%)a	84 (79%)a,b	39 (61%)c	0.004
Course, yrs	2.00 (1.00-3.75)	2.00 (1.00-4.00)	2.00 (1.00-4.00)	1.58 (1.00-4.00)	0.978
Clinical characteristic					
WBC (×10^9/L)	6.40 (5.04-8.38)	5.79 (4.48-6.89)a	6.28 (4.89-8.00)	5.75 (4.77-7.40)a	0.064
Hb (g/L)	125 (113-135)	123 (112-133)	127 (112-132)	127 (120-135)	0.261
PLT (×10^9/L)	256 (211-310)	261 (204-299)	244 (202-295)	257 (219-307)	0.678
ALT (U/L)	18 (14-27)	22 (14-68)	19 (11-28)	21 (11-49)	0.124
AST (U/L)	23 (14-38)	19 (14-59)	22 (12-38)	22 (11-61)	0.866
ESR (mm/h)	23 (12-40)	29 (16-57)a	34 (21-54)a	27 (17-51)a	<0.001
CRP (mg/h)	4 (1-13)	6 (2-15)	5 (2-10)	3 (1-17)	0.197
RF positive	178 (78%)	41 (72%)	84 (79%)	49 (77%)	0.773
CCP positive	187 (82%)	50 (88%)	99 (93%)a	57 (89%)	0.058
GPI positive	39 (17%)	7 (12%)	18 (17%)	10 (16%)	0.843
ANA					<0.001
ANA titre=0	121 (53%)	9 (16%)a	3 (2.8%)a	9 (14%)a,c	
0<ANA titre<=320	99 (43%)	35 (61%)a	76 (71%)a	46 (72%)a	
ANA titre>320	8 (3.5%)	13 (23%)a	28 (26%)a	9 (14%)a	
IgA (g/L)	2.68 (2.06-3.43)	2.53 (1.48-3.29)	2.54 (2.01-3.43)	2.79 (2.08-3.56)	0.222
IgM (g/L)	1.37 (1.02-1.95)	1.24 (0.93-1.75)	1.46 (0.93-2.05)	1.54 (0.97-1.90)	0.431
IgG (g/L)	14.4 (12.6-16.7)	17.4 (15.1-19.7)a	17.2 (14.4-19.6)a	14.9 (13.2-18.2)b,c	<0.001
C3 (g/L)	1.12 (0.99-1.30)	1.17 (0.99-1.30)	1.07 (0.94-1.28)	1.12 (1.00-1.31)	0.281
C4 (g/L)	0.26 (0.20-0.31)	0.25 (0.21-0.29)	0.24 (0.19-0.30)	0.24 (0.19-0.28)	0.488

Values are presented as median (interquartile range) for continuous variables and number (percentage) for categorical variables. Group comparisons were performed using the Kruskal–Wallis test for continuous variables and the Chi-square or Fisher’s exact test for categorical variables. Superscript letters indicate statistically significant pairwise differences as follows:

a Significantly different compared with the Ro52−/Ro60− group; b Significantly different compared with the Ro52+/Ro60− group; c Significantly different compared with the Ro52+/Ro60+ group. Statistical significance was set at p < 0.05.

BMI, body mass index; WBC, white blood cell count; Hb, hemoglobin; PLT, platelet count; ALT, alanine aminotransferase; AST, aspartate aminotransferase; RF, rheumatoid factor; CCP, cyclic citrullinated peptide; GPI, glucose-6-phosphate isomerase; ESR, erythrocyte sedimentation rate; CRP, C-reactive protein; IgA, immunoglobulin A; IgM, immunoglobulin M; IgG, immunoglobulin G; C3, complement component 3; C4, complement component 4.

### Disease activity and clinical outcomes

Patients with dual-positive antibodies (Ro52+/Ro60+) exhibited the highest disease activity among the four subgroups (**Table 3**). Specifically, DAS28-ESR scores were significantly higher in the Ro52+/Ro60+ group (median 4.97, IQR 4.15–6.33) compared to the Ro52−/Ro60− (median 4.39), Ro52+/Ro60− (median 4.57) and Ro52-/Ro60+ groups(median 4.55, p=0.002). Similarly, DAS28-CRP scores were elevated in the Ro52+/Ro60+ group (median 4.40) compared to the Ro52−/Ro60− group (median 4.09, p = 0.035).

**Table 3:** Association of anti-Ro52/Ro60 antibody profiles with disease activity and clinical outcomes.

Variable	Ro52-/Ro60- N = 228	Ro52+/Ro60- N = 57	Ro52+/Ro60+ N = 107	Ro52-/Ro60+ N = 64	P-value
DAS28-ESR	4.39 (3.53-5.46)	4.57 (3.55-5.60)	4.97 (4.15-6.33)a,b	4.55 (3.75-5.62)c	0.002
DAS28-CRP	4.09 (3.21-4.82)	4.16 (3.44-4.83)	4.40 (3.67-5.17)a	4.15 (3.09-5.29)	0.035
TJC28	5 (2-8)	6 (3-10)	5 (3-10)a	6 (4-12)	0.043
SJC28	2.0 (0.0-4.0)	2.0 (0.0-4.0)	4.0 (1.0-10.0)a,b	3.0 (1.0-6.0)a	<0.001
PtGA (100mm VAS)	50 (30-60)	50 (30-70)	50 (30-70)a,b	50 (30-65)c	0.327
PhGA (100mm VAS)	40 (30-50)	40 (30-60)	40 (30-50)	40 (30-50)	0.132
EQ-5D	0.69 (0.55-0.87)	0.68 (0.54-0.87)	0.76 (0.51-0.87)	0.68 (0.54-0.87)	0.972
HAQ-DI	0.63 (0.25-1.13)	0.75 (0.25-1.38)	0.88 (0.63-1.50)a	0.75 (0.38-1.44)	0.001
SDAI	25 (18-36)	29 (19-46)	25 (19-40)	29 (19-45)	0.247
CDAI	17 (11-24)	17 (13-26)	18 (13-26)a	20 (16-29)a	0.008
Limited range of motion	170 (75%)	38 (67%)	84 (79%)	49 (77%)	0.409
Morning stiffness	52 (23%)	22 (39%)a	35 (33%)	26 (41%)a	0.009
Joint deformity	41 (18%)	13 (23%)	35 (33%)a	17 (27%)	0.025
Functional impairment	8 (3.5%)	2 (3.5%)	0 (0%)	2 (3.1%)	0.164

Values are presented as median (interquartile range) for continuous variables and number (percentage) for categorical variables. Group comparisons were performed using the Kruskal–Wallis test for continuous variables and the Chi-square test for categorical variables. Superscript letters indicate statistically significant pairwise differences as follows:

a Significantly different compared with the Ro52−/Ro60− group; b Significantly different compared with the Ro52+/Ro60− group; c Significantly different compared with the Ro52+/Ro60+ group. Statistical significance was set at p < 0.05.

DAS28-ESR, Disease Activity Score 28 with Erythrocyte Sedimentation Rate; DAS28-CRP, Disease Activity Score 28 with C-Reactive Protein; TJC28, Tender Joint Count (28 joints); SJC28, Swollen Joint Count (28 joints); PtGA, Patient Global Assessment (100 mm Visual Analog Scale); PhGA, Physician Global Assessment (100 mm Visual Analog Scale); EQ-5D, EuroQol 5-Dimension Health Questionnaire; HAQ-DI, Health Assessment Questionnaire Disability Index; SDAI, Simplified Disease Activity Index; CDAI, Clinical Disease Activity Index.

Joint assessments also revealed more severe disease in the Ro52+/Ro60+ group, with significantly higher swollen joint counts (SJC28, median 4.0, p < 0.001) and tender joint counts (TJC28, median 5, p = 0.043) compared to the Ro52−/Ro60− group. Functional status, as measured by the Health Assessment Questionnaire Disability Index (HAQ-DI), was significantly worse in the Ro52+/Ro60+ group (median 0.88) compared to the Ro52−/Ro60− group (median 0.63,p = 0.001). In contrast, no significant differences were observed in patient global assessment (PtGA) or physician global assessment (PhGA) scores across the four groups.

While no significant differences in SDAI scores were noted, CDAI scores were significantly higher in the Ro52+/Ro60+ (median 18) and Ro52−/Ro60+ (median 20) groups compared to the Ro52−/Ro60− group (median 17, p = 0.008). Additionally, the prevalence of morning stiffness was significantly higher in the Ro52+/Ro60− (39%) and Ro52−/Ro60+ (41%) groups compared to the Ro52−/Ro60− group (23%, p = 0.009), while joint deformity was more common in the Ro52+/Ro60+ group (33%) compared to the Ro52−/Ro60− group (18%, p = 0.025).

### Risk of systemic complications

Patients with dual-positive anti-Ro52/Ro60 antibodies showed a markedly higher risk of several systemic complications, particularly sicca symptoms, interstitial lung disease (ILD), and hematologic involvement, compared to the double-negative group. These findings are summarized in **Table 4**, which presents the associations between anti-Ro52/Ro60 antibody profiles and the risk of various systemic complications. Specifically, the Ro52+/Ro60+ subgroup had an elevated risk of sicca symptoms (OR 2.49, 95% CI 1.06–5.94, p = 0.036) and ILD (OR 4.14, 95% CI 1.71–10.68, p = 0.002), with similarly increased odds of ILD seen in the Ro52+/Ro60− group (OR 4.49, 95% CI 1.58–12.78, p = 0.004). The association between Ro52−/Ro60+ and ILD was not statistically significant (OR 2.33, 95% CI 0.68–7.25, p = 0.151).

**Table 4:** Association of anti-Ro52/Ro60 Antibody profiles with systemic complications.

Variable	Group	OR_95%CI	P_value
Sicca symptoms			
	Ro52-/Ro60-	-	-
	Ro52-/Ro60+	2.04 (0.68–5.60)	0.177
	Ro52+/Ro60-	2.32 (0.77–6.40)	0.113
	Ro52+/Ro60+	2.49 (1.06–5.94)	0.036
Interstitial lung disease			
	Ro52-/Ro60-	-	-
	Ro52-/Ro60+	2.33 (0.68–7.25)	0.151
	Ro52+/Ro60-	4.49 (1.58–12.78)	0.004
	Ro52+/Ro60+	4.14 (1.71–10.68)	0.002
Hematologic involvement			
	Ro52-/Ro60-	-	-
	Ro52-/Ro60+	2.26 (0.74–6.33)	0.130
	Ro52+/Ro60-	2.56 (0.84–7.24)	0.081
	Ro52+/Ro60+	2.50 (1.02–6.19)	0.044
Renal involvement			
	Ro52-/Ro60-	-	-
	Ro52-/Ro60+	2.42 (0.31–14.91)	0.339
	Ro52+/Ro60-	2.73 (0.35–16.84)	0.278
	Ro52+/Ro60+	2.91 (0.63–15.01)	0.167
Liver involvement			
	Ro52-/Ro60-	-	-
	Ro52-/Ro60+	1.44 (0.20–6.85)	0.668
	Ro52+/Ro60-	2.48 (0.50–10.41)	0.224
	Ro52+/Ro60+	2.65 (0.78–9.38)	0.114

Odds ratios (ORs) with 95% confidence intervals (95% CIs) and p-values were calculated using logistic regression to assess the association between anti-Ro52/Ro60 antibody profiles and the presence of systemic complications, including sicca symptoms, interstitial lung disease (ILD), hematologic involvement, renal involvement, and liver involvement.

The Ro52−/Ro60− (double-negative) group was used as the reference category for all comparisons.Statistical significance was set at p < 0.05. No adjustments were made for covariates in these models.

Systemic complications were defined based on physician diagnosis or supporting clinical documentation at the time of RA evaluation.

Furthermore, the Ro52+/Ro60+ group was significantly associated with hematologic involvement (OR 2.50, 95% CI 1.02–6.19, p = 0.044), whereas no such association was detected for the Ro52+/Ro60− or Ro52−/Ro60+ subgroups. Associations with renal or hepatic involvement were not significant in any antibody group (all p > 0.05).Notably, both Ro52+/Ro60+ and Ro52+/Ro60− subgroups exhibited a significantly increased likelihood of ILD, while only the Ro52+/Ro60+ group was significantly associated with hematologic abnormalities. In contrast, no meaningful associations were observed for renal or liver involvement across any antibody profile.

### Therapeutic regimens and treatment response

In terms of treatment response, clinical remission(DAS28-ESR score <2.6) was significantly less frequent in the Ro52+/Ro60+ group (58%) compared to the Ro52−/Ro60− group (76%, p = 0.008). Furthermore, the prevalence of difficult-to-treat rheumatoid arthritis (D2T-RA) was significantly higher in the Ro52+/Ro60+ (13%) and Ro52+/Ro60− (11%) groups compared to the Ro52−/Ro60− group (3.5%, p = 0.006), suggesting a more refractory disease course in these antibody-positive subgroups. As shown in **Table 5**, the use of low-dose glucocorticoids (≤7.5 mg/day) was more common in the Ro52+/Ro60+ group (45%) compared to the Ro52−/Ro60− group (34%), though this difference was not statistically significant (p = 0.258). In terms of conventional DMARD use, patients in the Ro52+/Ro60+ group were more likely to have received more than two conventional DMARDs (33%) compared to the Ro52−/Ro60− group (19%, p = 0.031), with similar, though non-significant, trends observed in the Ro52+/Ro60− (28%) and Ro52−/Ro60+ (31%) groups. Prior biological DMARD use was significantly more common in the Ro52+/Ro60+ group (25%) compared to the Ro52−/Ro60− group (14%, p = 0.036), while targeted synthetic DMARD use did not differ significantly across the four subgroups (p = 0.280).

**Table 5:** Association of anti-Ro52/Ro60 antibody profiles with therapeutic regimens and treatment response outcomes.

Variable	Ro52-/Ro60- N = 228	Ro52+/Ro60- N = 57	Ro52+/Ro60+ N = 107	Ro52-/Ro60+ N = 64	P-value
Glucocorticoid ≤7.5mg	77 (34%)	23 (40%)	48 (45%)a	25 (39%)	0.258
Number of previous conventional DMARDs administered					0.031
≤2	184 (81%)	41 (72%)	72 (67%)a	44 (69%)	
>2	44 (19%)	16 (28%)	35 (33%)a	20 (31%)	
Previous biological DMARDs administered	31 (14%)	12 (21%)	27 (25%)a	8 (13%)	0.036
Previous targeted synthetic DMARDs administered	16 (7.0%)	7 (12%)	14 (13%)	7 (11%)	0.280
Clinical remission (DAS28-ESR<2.6)	173 (76%)	37 (65%)	62 (58%)a	42 (66%)	0.008
Clinical remission (DAS28-CRP<2.6)	170 (75%)	39 (68%)	71 (66%)	44 (69%)	0.412
Difficult-to-treat rheumatoid arthritis (D2T-RA)	8 (3.5%)	6 (11%)a	14 (13%)a	6 (9.4%)	0.006

Values are presented as median (interquartile range) for continuous variables and n (%) for categorical variables.

DMARDs, disease-modifying antirheumatic drugs. Statistical significance was set at p < 0.05.

a Significantly different compared with the Ro52−/Ro60− group.

### Difficult-to-treat rheumatoid arthritis

We first conducted a baseline comparison between the D2T-RA and non-D2T-RA groups and selected variables showing significant differences for inclusion in the multivariate analysis(**Supplementary Table 1**). In addition, RF and ACPA seropositivity were included based on prior literature supporting their relevance to RA disease severity and therapeutic response. Demographic covariates such as sex and BMI were also incorporated to account for potential confounding.

Subsequently, a multivariate logistic regression model was performed, with results presented in **Table 6**. The Ro52−/Ro60− (double-negative) group served as the reference. Patients with dual-positive Ro52+/Ro60+ antibodies exhibited the highest risk for developing D2T-RA (OR 3.61, 95% CI 1.39–10.00, p = 0.009), followed by those with Ro52+/Ro60− antibodies (OR 3.23, 95% CI 0.96–10.50, p = 0.058). Although patients with Ro52−/Ro60+ status had an elevated odds ratio (OR 2.71, 95% CI 0.80–8.71), the association did not reach statistical significance (p = 0.107).

**Table 6:** Risk of D2T-RA associated with Ro52/Ro60 antibody profiles.

Variable	OR (95% CI)	P-value
Ro52-/Ro60-	—	**0.043**
Ro52+/Ro60-	3.23 (0.96, 10.50)	
Ro52+/Ro60+	3.61 (1.39, 10.00)	
Ro52-/Ro60+	2.71 (0.80, 8.71)	
Male	0.20 (0.01, 1.13)	0.074
BMI (kg/m²)	0.85 (0.74, 0.97)	**0.014**
Alcohol consumption	4.54 (1.33, 13.70)	**0.018**
DAS28-ESR	1.14 (0.87, 1.48)	0.300
Anti-cyclic citrullinated peptide positive	1.44 (0.38, 9.40)	0.600
Rheumatoid factor positive	1.74 (0.66, 5.53)	0.300
Immunoglobulin G (g/L)	1.01(0.93, 1.07)	0.800
Interstitial lung disease	4.52 (1.65, 11.70)	**0.004**

Odds ratios (ORs) with 95% confidence intervals (CIs) and p-values were calculated using multivariate logistic regression with backward stepwise elimination (retention threshold: p < 0.05). The outcome of interest was difficult-to-treat rheumatoid arthritis (D2T RA). The anti-Ro52−/Ro60− (double-negative) group served as the reference for antibody subgroup comparisons. Covariates included sex, BMI, alcohol consumption, DAS28-ESR, RF and ACPA seropositivity, serum IgG levels, and the presence of interstitial lung disease (ILD). Statistically significant associations (p < 0.05) are indicated in bold.

Among other covariates, lower BMI was independently associated with a reduced risk of D2T-RA (OR 0.85, 95% CI 0.74–0.97, p = 0.014). Alcohol consumption (OR 4.54, 95% CI 1.33–13.70, p = 0.018) and the presence of interstitial lung disease (ILD; OR 4.52, 95% CI 1.65–11.70, p = 0.004) were significant risk factors. Sex, DAS28-ESR, RF, ACPA, and IgG levels were not independently associated with D2T-RA in the final model.

**Figure 2:**
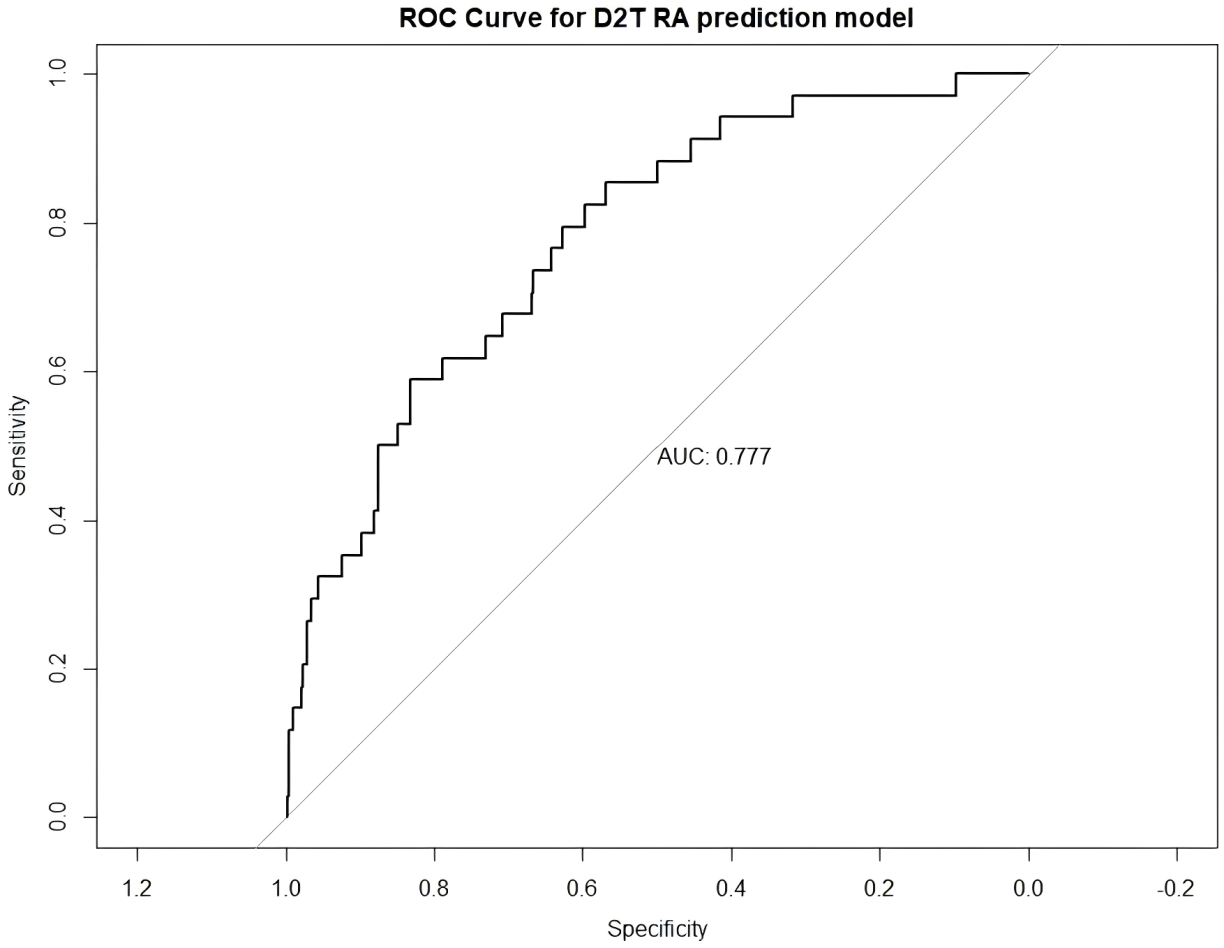
ROC curve of the multivariable prediction model for difficult-to-treat rheumatoid arthritis (D2T RA). The model demonstrated good discriminatory ability with an area under the curve (AUC) of 0.777.

To evaluate the discriminative performance of the final logistic regression model, we generated a receiver operating characteristic (ROC) curve (**Figure 2**). The model demonstrated good predictive ability, with an area under the curve (AUC) of 0.777, indicating acceptable discrimination between D2T-RA and non-D2T-RA patients.

## Discussion

In this study, we conducted a comprehensive evaluation of the clinical and immunological profiles of rheumatoid arthritis (RA) patients stratified by anti-Ro52 and anti-Ro60 antibody status. Our analysis revealed marked heterogeneity in disease activity, systemic involvement, therapeutic response, and long-term outcomes across these subgroups, highlighting the importance of precise antibody profiling for personalized disease management. Patients with dual-positive (Ro52+/Ro60+) antibodies exhibited the most severe disease phenotype, characterized by higher disease activity, more frequent systemic complications, and a significantly increased risk of developing difficult-to-treat RA (D2T-RA). In contrast, patients with isolated Ro60 positivity generally had a milder disease course, with fewer systemic manifestations and lower IgG levels, yet still displayed elevated erythrocyte sedimentation rates (ESR) and moderate disease activity, indicating persistent inflammatory activity.

Anti-SSA/Ro antibodies, comprising Ro60 and Ro52 isoforms, are increasingly recognized as distinct molecular entities encoded by separate genes and localized to divergent cellular compartments (22, 23). While anti-Ro52 is more prevalent in Sjögren’s disease (SjD) and systemic lupus erythematosus (SLE) (14, 24), its lower frequency in RA may contribute to under recognition of this phenotype. Previous RA studies mainly focused on anti-Ro positivity (21) or coexistence of secondary SjD and RA (25), identifying a subgroup characterized by female predominance, elevated B-cell activation markers, and attenuated responses to conventional therapies (26). Our findings refine this paradigm by demonstrating that dual anti-Ro52/Ro60 positivity—rather than isolated anti-Ro60 reactivity—drives the association with refractory disease, suggesting prior studies may have overlooked critical heterogeneity within anti-Ro-positive RA populations.

The association between dual antibody positivity and severe RA manifestations may be mediated by immune mechanisms involving type I IFN activity. In SLE (27) and SjD (13), dual anti-Ro52/Ro60 positivity correlates with heightened IFN signatures, hypergammaglobulinemia, and systemic damage—phenotypes mirroring our observations in RA. Gene expression analyses reveal a gradient of IFN-α activity aligned with anti-Ro profiles, with dual-positive individuals exhibiting maximal IFN pathway activation (28). Given IFN-α’s role in perpetuating synovial inflammation (29) and impairing treatment responses (30), this mechanism may underpin the aggressive phenotype observed in dual-positive RA. Supporting this, elevated serum IFN-α levels in RA correlate with increased cardiovascular risk (31) and inflammatory burden (32–34), while IFN-related gene signatures predict responses to biologics. Future studies directly interrogating IFN activity in dual-positive RA cohorts are warranted to validate this hypothesis and explore therapeutic targeting of this pathway.

These findings align with emerging evidence across autoimmune diseases. In SjD, dual anti-Ro52/Ro60 positivity predicts severe glandular dysfunction and systemic manifestations, whereas isolated anti-Ro60 associates with milder phenotypes (35). Similarly, SLE patients with concurrent anti-Ro52/Ro60 exhibit higher disease activity and therapeutic demands (14, 36). Clinically, this underscores the need for autoantibody subtyping to optimize risk stratification and therapeutic decision-making.

A key challenge in RA management is the early identification of patients likely to develop difficult-to-treat RA (D2T RA) (18). Prior studies have linked D2T RA with factors such as older age, high RF titers, elevated disease activity, low methotrexate dosage, and comorbidities like hypertension and diabetes (19). A French cohort also reported associations with low socioeconomic status, ILD, and higher DAS28-CRP scores (37). However, most of this research has focused on clinical predictors, with little attention to immunological biomarkers.

Our study helps fill this gap by identifying anti-Ro52/Ro60 antibody profiles—particularly dual positivity—as potential predictors of D2T RA. These autoantibodies may reflect underlying immune dysregulation and offer a practical tool for early risk stratification. Incorporating antibody profiles into routine assessment could improve treatment planning and outcomes. Further prospective studies are needed to validate these findings and explore the mechanisms linking Ro antibodies to treatment resistance. Several limitations of our study should be acknowledged. This was a single-center, cross-sectional analysis, which limits the generalizability of the findings and precludes any assessment of causality or long-term outcomes. Another limitation is the lack of direct measurement of type I interferon levels or gene signatures in our patients. Finally, we did not analyze certain biomarkers that could further illuminate the mechanistic link between Ro antibodies and RA pathogenesis. These gaps should be addressed in future investigations.

In conclusion, concurrent positivity for anti-Ro52/TRIM21 and anti-Ro60/SSA antibodies delineates a clinically distinct and immunologically unique RA subgroup characterized by increased disease severity and therapeutic complexity. Future longitudinal and mechanistic studies are essential to comprehensively validate these findings and support the development of targeted therapeutic approaches tailored to this challenging subgroup of RA patients.

## Data Availability

The data that support the findings of this study will be made available by the authors upon reasonable request.
